# Oral administration of recombinant *Bacillus subtilis* spores expressing mutant staphylococcal enterotoxin B provides potent protection against lethal enterotoxin challenge

**DOI:** 10.1186/s13568-020-01152-x

**Published:** 2020-12-14

**Authors:** Zhile Xiong, Jialiang Mai, Fei Li, Bingshao Liang, Shuwen Yao, Zhuwei Liang, Chao Zhang, Fei Gao, Xiaolan Ai, Jielin Wang, Yan Long, Min Yang, Sitang Gong, Zhenwen Zhou

**Affiliations:** 1 Clinical laboratory, Guangzhou Women and Children’s Medical Center, Guangzhou Medical University, Guangzhou, 510623 Guangdong China; 2 Department of Gastroenterology, Guangzhou Women and Children’s Medical Center, Guangzhou Medical University, Guangzhou, 510623 Guangdong China

**Keywords:** *Staphylococcus aureus*, Staphylococcal enterotoxin B, Vaccination, *Bacillus subtilis*

## Abstract

Pathogenicity of *Staphylococcus aureus* is induced by staphylococcal enterotoxin B (SEB). A mutant form of SEB (mSEB) is immunogenic as well as less toxic. Recombinant mSEB and SEB were expressed in pET28a prokaryotic plasmids. Tumor necrosis factor-α (TNF-α) and interleukin-6 (IL-6) levels in mSEB-stimulated macrophages were lower than those in SEB-stimulated macrophages (*p* < 0.001, *p* < 0.01 respectively). Using CotC as a fusion protein, we constructed recombinant *Bacillus subtilis* spores expressing mSEB on the spore surface and evaluated their safety and protective efficacy via mouse models. Oral administration of mSEB-expressing spores increased SEB-specific IgA in feces and SEB-specific IgG1 and IgG2a in the sera, compared with mice in naïve and CotC spore-treated groups (*p* < 0.001, *p* < 0.01, *p* < 0.001 respectively). Six weeks following oral dosing of recombinant spores, significant differences were not found in the serum biochemical indices between the mSEB group and the naïve and CotC groups. Furthermore, oral administration of mSEB spores increased the survival rate by 33.3% in mice intraperitoneally injected with 5 µg of wild-type SEB plus 25 µg lipopolysaccharide (LPS). In summation, recombinant spores stably expressing mSEB were developed, and oral administration of such recombinant spores induced a humoral immune response and provided protection against SEB challenge in mice.

## Key points

- Mutant staphylococcal enterotoxin B induced less cytokine secretion in macrophages compared with non-mutant one.
- Recombinant *B. subtilis* spores expressing mutant staphylococcal enterotoxin B induced specific mucosal and systemic immunity.
- Oral administration of Recombinant *B. subtilis* spores expressing mutant staphylococcal enterotoxin B could provide protection against enterotoxin B challenge in mice.

## Introduction

*Staphylococcus aureus* is a common, highly pathogenic bacterium that causes severe suppurative infection and endangers human health globally (Lakhundi and Zhang 2018). Accumulation of virulent factors produced by *S. aureus* may contribute to self-limiting gastrointestinal intoxication leading to toxic shock, a potentially fatal syndrome (Asensi et al. 2013; Chen et al. 2019). Staphylococcal enterotoxin B (SEB), one of the most prototypical enterotoxins (Anderson et al. 2012), binds directly to human major histocompatibility complex class II (MHC II) molecules. The bimolecular complex of SEB-MHC II subsequently links up with the T cell receptor Vβ chain, resulting in a ternary complex (Quiel et al. 2010). Once formed, the trimolecular complex triggers T cells to produce a cytokine storm, which may contribute to SEB-mediated symptoms or even life-threatening toxic shock (Baker et al. 2002; Choi et al. 2017). In addition, SEB functions as a superantigen which stimulates monocyte-macrophage secreted cytokines and augments the proliferation of SEB-induced T cells (Szabo et al. 1995). Moreover, harsh environmental conditions such as proteolytic degradation, heat, and changes in pH, do not affect the structure of SEB (Choi et al. 2017). Such characteristics allow SEB to pose a major threat to anti-biological warfare or anti-bioterrorism agents. Therefore, development of immunotherapeutics or vaccinations against SEB is urgently desired.

Molecular and physiological studies that investigated the structure-function relationships of SEB (Ulrich et al. 1998) have compiled a large amount of data that define the relationships of the toxin, SEB L45 Y89 Y94, indicating that it plays a key role in MHC II and TCR binding (Ulrich et al. 1998). Various vaccination regimens have demonstrated that mSEB (L45R, Y89A, Y94A) exhibits antigenic structures which are devoid of toxicity and elicit protective immune effects against wild-type SEB challenge in mice and rhesus monkeys. This indicates that mSEB may be considered as a candidate molecule for a potential SEB vaccine (Boles et al. 2003; Choi et al. 2017; Inskeep et al. 2010; Liu et al. 2019).

*Bacillus subtilis* is a naturally occurring gram-positive microorganism which possesses probiotic properties beneficial to humans (de Souza et al. 2014). In a previous study of ours, we constructed a recombinant *B. subtilis* surface, expressing cholera toxin B (CTB)-conjugated *Helicobacter pylori* urease protein B (UreB) which provided protection against *H. pylori* infection(Zhou et al. 2017). The current study assessed the cytokine reactivity of mSEB cultured with human macrophages in vitro, using *B. subtilis* as a delivery vaccine vehicle that carries mSEB, a candidate antigen for vaccination against SEB. Furthermore, we evaluated its safety and protective efficacy in mice.

## Materials and methods

### Mice

Six-week old female specific pathogen-free BALB/C mice were purchased from Guangdong Medical Laboratory Animal Center (Foshan, China). Mice were maintained under special pathogen-free conditions and fed laboratory chow and water. All experiments involving animals were approved by the Animal Experiments Committee of Guangzhou Women and Children’s Medical Center.

### PCR amplification of *mSEB* gene and purification of mSEB protein

For the purposes of this study, the *mSEB* (L45R, Y89A, Y94A) nucleotide sequences were synthesized by Beijing Genomics Institute (Shenzhen, China). Subsequently, *mSEB* was amplified via polymerase chain reaction (PCR). The primers utilized were as follows: forward primer (5‘-aaagagctcgagagtcaaccagatccta-3’) and a reverse primer: (5‘-agactcgagtcactttttctttgtcgtaagat3-’) with restriction sites for *Sac*I and *Xho*I (underlined), allowing amplified DNA to be cloned into a pET28a expression plasmid (Merck, Germany) and transformed into *Escherichia coli* BL21. All recombinant plasmids were identified via DNA sequencing. The *mSEB* sequences have been submitted to GenBank (accession no. MT937070).

The mSEB proteins were produced via isopropylb-D-thiogalactopyranoside (IPTG) induction using pET28a-mSEB *E. coli*, as previously described (Zhou et al. 2008). The fusion protein was purified using a Ni-NTA resin column (Sangon Biotech, China) and Detoxi-Gel™ Endotoxin Removing Gel (Thermo, USA). Next, mSEB purified protein was verified using SDS-PAGE and western blotting.

### Surface expression of recombinant mSEB on *Bacillus subtilis* spores

We obtained *mSEB* by subjecting the pET28a-mSEB plasmid, constructed during the previous step, to a PCR. The primers for PCR contained restriction sites for *Xba*I and *Pst*I (underlined), and were as follows: forward primer, (5‘-aaatctagagagagtcaaccagatccta-3’); and reverse primer (5‘-aaactgcagtcactttttctttgtcgtaagat-3’). After double restriction enzyme digestion with *Xba*I and *Pst*I, the fragments were cloned into the plasmid pUS186-CotC (Zhou et al. 2008) at the 3’ terminal of CotC. The recombinant plasmid was sequenced and transformed into *B. subtilis* WB600 cells. Sporulation of recombinant *B. subtilis* was induced via Difco sporulation medium (DSM) as previously described (Mai et al. 2019). Spores were centrifuged at 8000 × g for 15 min, and the supernatant was removed. Following resuspension with PBS, 4 mg/mL lysozyme was added to the precipitate, which was then incubated at 35 °C for 15 min to remove residual sporangial cells. Next, spores were collected and washed initially with 1 M NaCl and 1 M KCl, and then washed twice in distilled water. Finally, the spores were resuspended in sterile phosphate buffer saline (PBS), and the residual propagules were removed by incubating the spores at 68 °C for 1 h. Spore numbers were counted using a hemocytometer, and recombinant coat proteins were verified using SDS-PAGE and western blotting (Fig. 2).

**Fig. 1:**
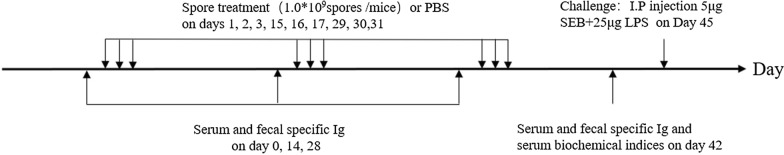
Immunization and challenge protocol for experiment mice. Mice were treated with 10^9^ spores on days 1, 2, 3, 15, 16, 17, 29, 30, and 31 and blood and stool samples were collected for specific Ig measurement on days 0, 14, 28 and 42 respectively. On day 45, mice were challenged with 5 µg SEB plus 25 µg LPS by intraperitoneally injection

### Macrophage differentiation and culture with SEB and mSEB

Peripheral blood mononuclear cells (PBMCs) were isolated from human peripheral blood using Ficoll-Hypaque solution via density-gradient centrifugation. Next, monocytes were selected from PBMCs using CD14 + microbeads (Miltenyi Biotec, USA) and incubated with 10% human type AB plasma for the purpose of macrophage differentiation (Netea et al. 2009). After being cultured for 5 days, cells were stimulated using 5 µg/ml SEB, 5 µg/ml mSEB, and 5 µg/ml LPS as a positive control. Culture supernatants were harvested 24 h later, upon which resultant interleukin-6 (IL-6) and tumor necrosis factor-α (TNF-α) were quantified using an ELISA kit (BD Biosciences, USA).

### Oral delivery of recombinant spores and sample collection

Eighteen mice were divided into three groups of six mice each, where in the control group contained naïve mice. Mice in the CotC group were orally administered 1.0 × 10^9^ spores expressing CotC, while the mice in the mSEB group were orally administered 1.0 × 10^9^ spores expressing mSEB on days 1, 2, 3, 15, 16, 17, 29, 30, and 31 (administered on three consecutive days every 2 weeks), as depicted in Fig. 1. Naïve mice were administered PBS at the same time. Serum and feces samples were collected on days 0, 14, 28 and 42, and stored at − 80 °C until required for further analyses. Fecal samples were treated as described previously (Zhou et al. 2008).

**Fig. 2:**
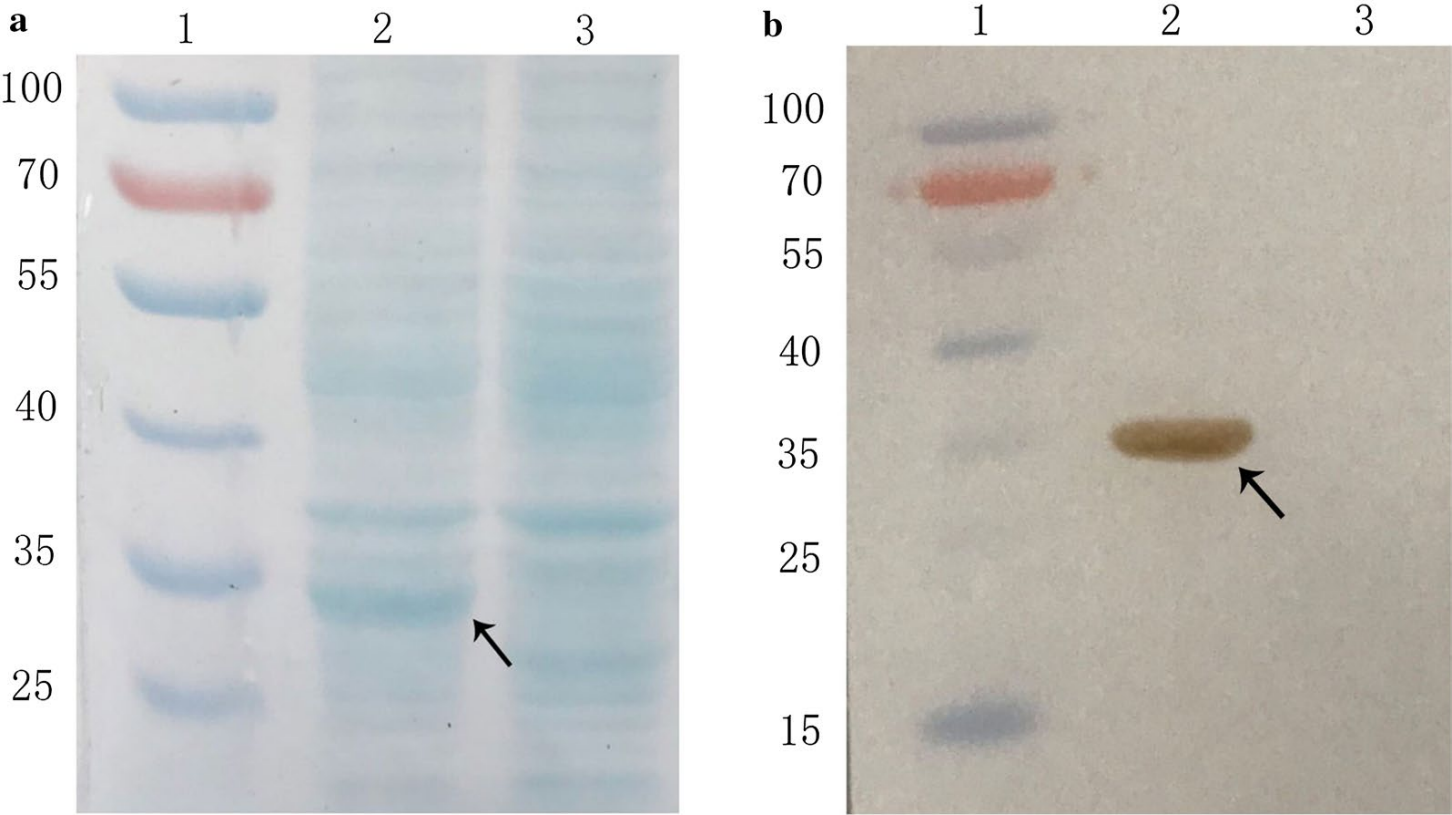
SDS-PAGE and Western blot of recombinant mutant Staphylococcal enterotoxin B in *E. col*i BL21. **a **SDS-PAGE (12%) of mSEB in *E. coli* BL21. Lane 1: prestained protein markers, Lane 2: lysate of the bacterial with recombinant plasmid pET28a-mSEB induced with IPTG, lane 3: lysate of the bacterial with parent pET28a plasmid induced with IPTG. **b **Western blot of mSEB in *E.coli* BL21. Lane 1: prestained protein markers, Lane 2: lysate of the bacterial with recombinant plasmid pET28a-mSEB, Lane 3: lysate of the bacterial with parent pET28a plasmid; The arrows indicate the target protein

### Specific antibody secretion assays

SEB-specific secretory IgA (sIgA) in the feces and serum SEB-specific IgG1 and IgG2a were measured using ELISA. Microplates were coated with 100 µL of purified SEB (5 µg/mL) and incubated overnight at 4 °C. The plates were washed five times with PBST and blocked with 10% fetal bovine serum (FBS, Ruite Biotechnology Co. Ltd., Guangzhou, China) for 2 h at 37 °C, following which 100 µL of fecal supernatant or diluted serum (1:4000) were added to each well; the plates were then incubated at 4 °C overnight. On the second day, the plates were incubated with HRP-conjugated goat anti-mouse sIgA (1:5000, Southern Biotech, Birmingham, USA), IgG1 (1:5000, Southern Biotech), or IgG2a (1:5000, Southern Biotech), respectively, for 2 h at 37 °C. Next, the plates were washed five times with PBST. Finally, 100 µL of tetramethylbenzidine (TMB) substrate (KHB, Shanghai, China) and 50 µL of stop buffer (KHB) were added. The resulting signals were detected at 450 nm.

### Detection of biochemical indices

In order to evaluate the safety of orally administrated spores in mice, relevant biochemical indices in the sera of mice were measured on the sixth week after oral dosing with recombinant spores, using an automated clinical chemistry analyzer (Beckman Coulter AU5811, USA). The relevant index was defined by the activities of the following: alanine aminotransferase (AST); aspartate aminotransferase (AST); serum creatinine (CR); serum urea (URE); creatine kinase (CK); and creatine kinase MB (CK-MB).

### SEB challenge

Two weeks after last orally administration of recombinant spores, mice in the three groups were intraperitoneally injected with 5 µg of SEB and 25 µg of LPS, following which survival was recorded 12, 24, 36 and 48 h later to determine the protection status of mSEB vaccine spores.

### Statistical analysis

Data are expressed as mean ± standard deviation (SD). Statistical analyses were performed using analysis of variance (ANOVA). All tests were two tailed, and statistical significance was set at *p* < 0.05. Data were analyzed using SPSS for Windows, version 16.0.

## Results

### Expression of mSEB in *Escherichia coli*

Recombinant plasmids (pET-28a-mSEB) containing the mutant *S. aureus* enterotoxin B were constructed. The entire *mSEB* fragment was amplified to the expected size (approximately 720 bp) from purified pET-28a mSEB plasmid and the recombinant mSEB plasmid was verified via double-enzyme digestion and sequencing. Following induction with 0.5 mM IPTG, SDS-PAGE and western blot results show that a major band which had an estimated molecular weight of 27.8 kDa was expressed (Fig. 2). In addition, we expressed a SEB protein in an estimated molecular about 27.8 kDa from the pET28a-SEB plasmid which was previously constructed(Li et al. 2016). 1 mg/mL SEB and mSEB protein was purified though Ni-NTA column and Detoxi-Gel™ Endotoxin Removing Gel (with endotoxin ≤ 0.1 EU/µg). SEB and mSEB proteins were stored at − 80 °C for further study.

### Expression of mSEB in *Bacillus subtilis* spore coats

**Fig. 3:**
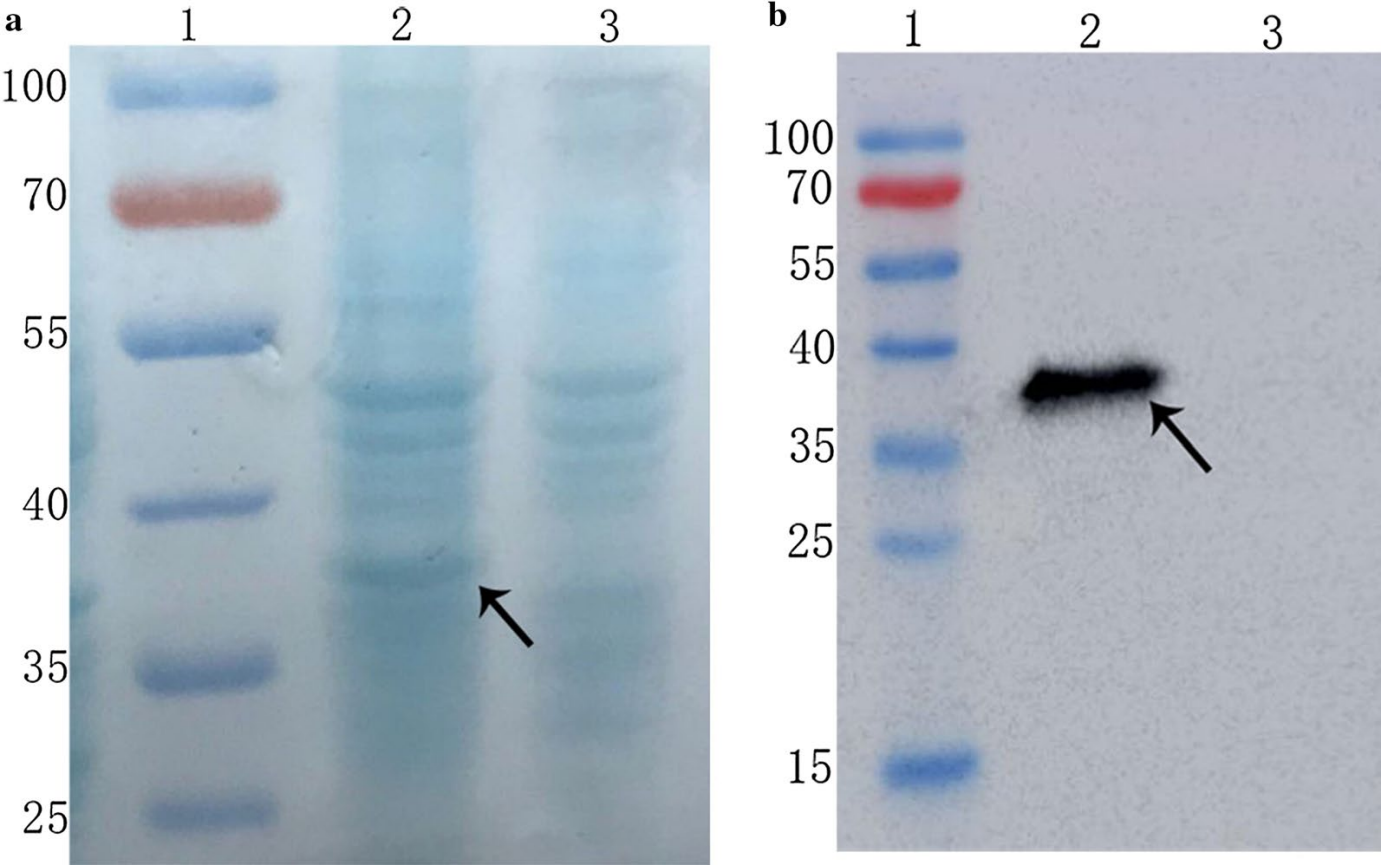
SDS-PAGE and Western blot of recombinant mutant Staphylococcal enterotoxin B in *B. subtilis* spores. **a** SDS-PAGE analysis of coat proteins extracted from recombinant mSEB spores. Lane 1, prestained protein marker; Lane 2, mSEB spore coat proteins; lane 3, CotC spore coat proteins; **b** Western blot analysis of coat proteins extracted from recombinant mSEB spores. Lane 1, prestained protein marker; lane 2, mSEB spore coat proteins; lane 3, CotC spore coat proteins; The arrows indicate the target protein

*mSEB* DNA fragment of about 720 bp was amplified by PCR using the plasmid pET28a-mSEB as template. Then *mSEB* was sub-cloned to plasmid pUS186CotC, followed by transformed into *B. subtilis* WB600, sporulation was induced by nutrition exhausting method, about 10^11^ recombinant mSEB spores were obtained from 1L DSM. The spores coat protein was extracted in SDS-DTT buffer via sonication, and 12% SDS-PAGE analysis showed that the recombinant CotC-mSEB protein was approximately 36.6 kDa (8.8 kDa CotC + 27.8 kDa mSEB) (Fig. 3a). As presented in Fig. 3b, western blotting with SEB-specific antisera also revealed positive bands in the spores coat, indicating the successful expression of heterologous recombinant mSEB protein on the spore surface.

### Macrophages secreted less IL-6 and TNF-α upon incubation with mSEB

**Fig. 4:**
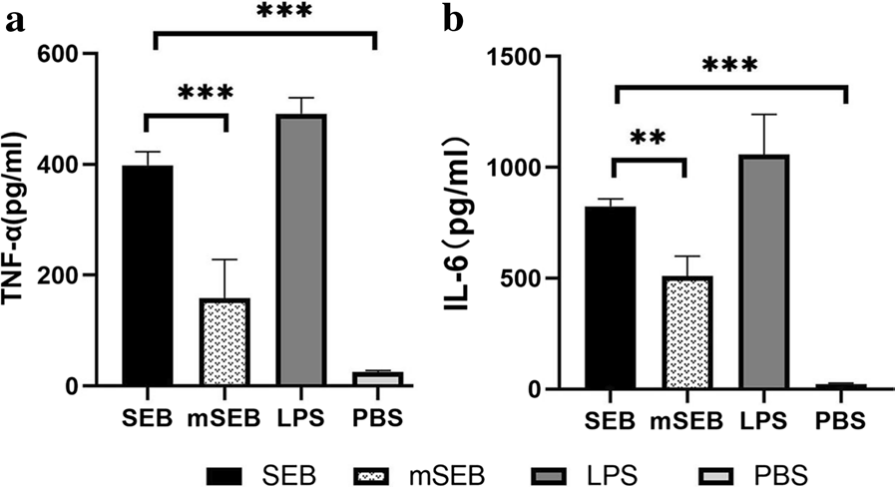
Detection of TNF-a and IL-6 level in macrophage stimulated with mSEB and SEB. Cytokine was determined using ELISA after 24 h coculture of human macrophage with SEB, mSEB and LPS **a** TNF-alevel, **b **IL-6 level. *p < 0.05, ** *p* < 0.01. ***p < 0.001

SEB stimulate macrophages in the absence of T-cells to secrete cytokines such as IL-6 and TNF-α, which, in turn, augment a T-cell induced cytokine storm. mSEB is a mutation of SEB residues (L45, Y89, Y94) necessary to bind to the MHC II. In order to evaluate the cytokine responses of human macrophages to mSEB, we extracted CD14 + monocytes from healthy individuals and differentiated those monocytes into macrophages by adding 10% pooled human plasma. The macrophages were quite less responsive to 5 µg/ml mSEB, as compared with 5 µg/ml of SEB which stimulate a higher TNF-α and IL-6 response. As showed in Fig. 4, TNF-α level in SEB stimulated-macrophage was 3 time higher than the mSEB-stimulated macrophage, while IL-6 levels in SEB stimulated macrophage was 1.5 times higher than mSEB stimulated macrophage. These in vitro studies indicated that macrophages react less with mSEB compared with SEB.

### Recombinant mSEB spores induced mucosal and systemic immunity

**Fig. 5:**
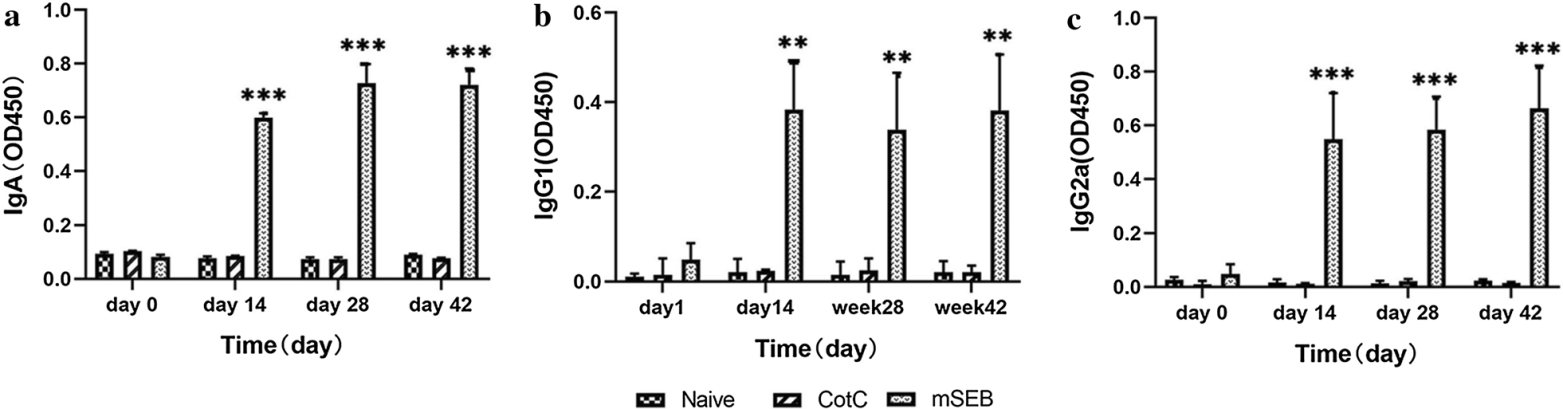
Detection of SEB-specific antibodies in mice after oral administration with spores. SEB-specific antibodies level from mice intragastrically gavage with PBS (naïve group), CotC spores (CotC group), and mSEB spores (mSEB group) at day 0, 14, 28, and 42. Results are presented as mean values and standard deviations (SD) **a** Fecal SEB-specific IgA level, **b** serum SEB-specific IgG1 level, **c** serum SEB-specific IgG2a level. *p < 0.05, **p < 0.01, ***p < 0.001

To assess SEB-specific antibody level from colorectal secretions and serum, we assessed fecal and serum samples from 2 weeks after oral immunization (weeks 2, 4, 6) by ELISA. Consistent with our previous study(Zhou et al. 2015), antigen specific IgA and IgG responses were induced in both the mucosa and serum. Two weeks after the mice were treated with recombinant spores, a significant increase in SEB-specific IgA levels was observed in the feces of mSEB spore-treated mice compared with those in the naïve and CotC groups (*p* < 0.001) (Fig. 5a). On day 28, the SEB-specific IgA in the feces of the mSEB spore-treated group reached a peak. On the day prior to the challenge (day 42), sIgA in the mSEB spore-treated group remained at a higher level, at 6-fold higher than those of the naïve and CotC groups. There were no significant differences between the sIgA levels of the CotC group and the naïve group. Two weeks following immunization, serum levels of anti-SEB IgG1 and IgG2a were significantly increased in mice orally administered mSEB spores compared with those in the other groups (*p* < 0.01, *p* < 0.001 respectively). High levels of SEB-IgG1 and IgG2a were observed on the day prior to the challenge (day 42) (Fig. 5b, c). No significant differences were observed in the anti-SEB IgG1 and IgG2a levels between the naïve and CotC groups. This indicated that oral dosing with mSEB spores induced both mucosal and systemic immunity.

### Serum biochemical indices

**Fig. 6:**
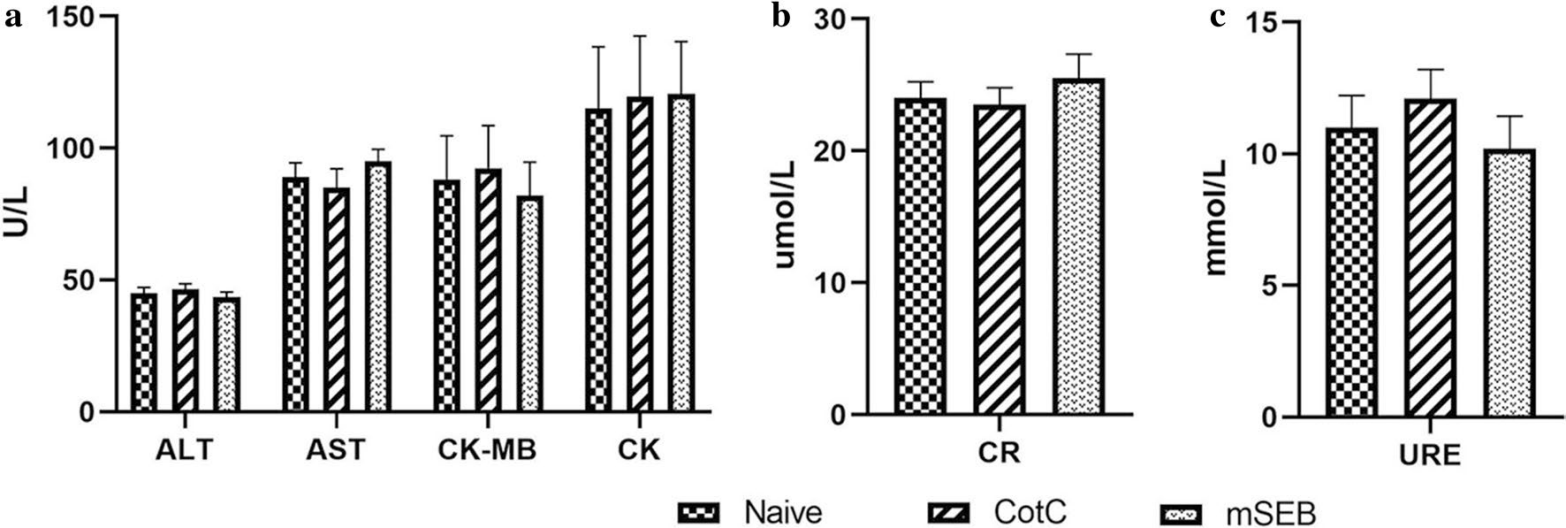
The biochemical parameters in serum. After 6 weeks treatment, the biomedical indices of ALT, AST, CK-MB, CK, CR and URE in serum was analyzed by automated clinical chemistry analyzer. **a** ALT, AST, CK-MB and CK level, **b** CR level, **c** URE level

In order to study the safety of orally dosing recombinant spores, the serum levels of ALT, AST, CK CK-MB, CR, and URE were studied on week 6 (Fig. 6). Liver function-related indices (ALT and AST) in the sera of naïve and CotC groups remained at levels that were similar to those in the mSEB spore group. Meanwhile, kidney and cardiac-related indices (CR, URE, CK, and CK-MB) of the mSEB spore group (*p* > 0.05) were not significantly different from those in the naïve and *B. subtilis* CotC groups. This indicated that oral dosing with mSEB spores did not affect the myocardial enzymes, liver and kidney function in mice, which showed good safety records of recombinant mSEB spores.

### Protection of BABL/c treated with mSEB spores

**Fig. 7:**
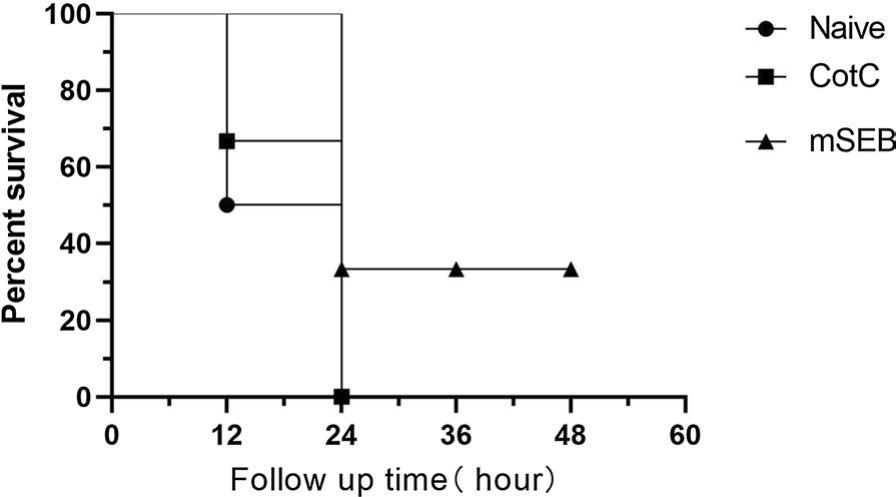
Survive rates in mice after SEB challenge. Two weeks after the last oral administration of mSEB spores, mice were challenged intraperitoneally with 5 µg SEB and 25 µg LPS, and the survive rates were recorded in 12, 24, 36, 48 h to determine the protection efficacy of mSEB spores

Although mSEB spores induced an effective specific immune response in mice, their protection against SEB challenge required further study. The protective efficacy of mSEB spores was investigated in classical mouse BALB/c SEB toxin challenge models. Two weeks after the final oral administration of mSEB spores, the mice were challenged with 5 µg SEB and 25 µg LPS. The results showed that 66.7% of mice in the CotC group and 50% of mice in the naïve group survived within 12 h while 100% of the mice survived within 12 h in the mSEB treated group. On 24 h of observation, 33.3% of the mice treated with mSEB spores survived, while the naïve group and CotC group mice died within 24 h following the challenge (Fig. 7). These results suggest that mSEB spores can provide protective effect on lethal enterotoxin challenge.

## Discussion

Staphylococcal enterotoxin B is a common infective agent which is associated with food poisoning and toxic shock (Karauzum et al. 2012). Exposure to SEB may cause vomiting, fever, toxic shock syndrome, immunosuppression, wasting and chronic joint inflammation (Argudín et al. 2010). However, legislated vaccines or other effective therapeutic agents that protect against SEB are currently lacking (Liu et al. 2019). Moreover, SEB which is excreted by *S. aureus* and can contaminate food sources, poses the threat of being utilized as a lethal biological weapon (Burnett et al. 2005). Therefore, the need for an effective vaccine against SEB is urgently required.

Presently, mSEB is considered a suitable candidate molecule for a nontoxic SEB vaccine which induces a vigorous immune response. Previous studies have shown that mSEB (L45R, Y89A, and Y94A) is unable to bind to MHC class II molecules, and lacks the ability to activate T-cell expansion (Boles et al. 2003). SEB can function as a superantigen that stimulates antigen-presenting cells (APCs) such as macrophages, which secrete cytokines that augment T-cell proliferation in order to transmit downstream signals (Palkama and Hurme 1993). SEB infection is associated with the induction of TNF-α and other pro-inflammatory cytokines (Korolev et al. 2003). The current study incubated human macrophages with SEB as well as mSEB. The results indicated that SEB induced monocyte-macrophage production and secretion of TNF-α and IL-6, whereas mSEB stimulated macrophages produced less TNF-α and IL-6. This indicated that mSEB reacts less with macrophages and may possibly be less toxic than SEB. This result suggests that mSEB is a safe vaccine candidate.

Our previous study successfully delivered heterologous proteins, such as UreB and CTB-NAP, to *B. subtilis* surfaces (Dong et al. 2017; Zhou et al. 2015). The current study used recombinant methods to construct a recombinant plasmid expressing mSEB, which was transformed into *B. subtilis* WB600 using Spizizen competent transformation. Based on the bicinchoninic acid protein quantitation assay (BCA), 0.021 pg of coat proteins can be isolated from each spore. We compared the gray value in Fig. 3a using Image J v1.80 software and speculated that the amount of SEB isolated from 1.0 × 10^9^ recombinant spores was about 1100 ng (data not shown). SEB-specific antibodies in mice sera and feces were significantly increased following two weeks of treatment with mSEB spores (3 times a week). This result suggests that mSEB retains strong immunogenicity on the recombinant spore surface. Moreover, recombinant spores are stable in the gastrointestinal tract and effectively stimulate the body to produce specific antibodies without assistance from other adjuvants. The results showed that orally administering mSEB spores produced higher titers of SEB-specific IgA in the feces as well as higher SEB-specific IgG1 and IgG2a levels in the sera, compared to those in the naïve group and CotC group. Fecal IgA functions as an antibody secreted by mucosal immune response. Toxins at the surface of the mucosa are neutralized by secretory IgA, engulfed in mucus, and cleared via peristaltic and mucosal activity. This promotes the clearance of pathogenic microorganisms and antigens (Mantis et al. 2011). The current study indicated that SEB-specific IgA levels were significantly increased following the two weeks of immunization. Moreover, administering mSEB spores orally induced both Th1 and Th2 immune responses. IgG2a antibody response usually represents helper T(Th)1-mediated humoral immunity, whereas IgG1 represents Th2-mediated immunity in mice. Our results showed that both SEB-specific IgG1 and IgG2a in the mSEB spore-treated group were higher than those in the other groups.

Safety is a major consideration in vaccine development. SEB functions as a superantigen that causes multi-organ failure and death at low concentrations. ALT and AST, which mainly reside in hepatocytes, are critical indicators of liver function. When hepatocytes are damaged, ALT and AST are released into the blood (Gao et al. 2017). In addition, serum CR and URE are important indicators of kidney injury (Islam et al. 2019), whereas CK and CK-MB are associated with cardiac inflammation and function (Gao et al. 2019). We fused *mSEB* with the outer coat protein of *B. subtilis*, CotC, and observed that there was no significant difference between the biochemical enzymatic activities of the mSEB group and those of the naïve and CotC groups. The average body weights of the CotC, mSEB, and naïve groups, prior to the challenge, were not significantly different (*p* > 0.05, data not shown), indicating that mSEB spores were not harmful to health.

Reportedly, intraperitoneal injection of SEB and LPS into mice is a widely accepted lethal SEB model (Boles et al. 2003; Choi et al. 2017; Liu et al. 2019). Mice treated with mSEB spores were challenged with 5 µg of SEB and 25 µg LPS, and the challenge test of mice treated with mSEB showed that mSEB spores produced a partial protective effect (33.3%) against SEB. This may be due to the quantity of recombinant *B. subtilis* spores used and the duration of spore treatment not being sufficient for the mice to develop full protection against the SEB challenge. Therefore, the experimental dose of *B. subtilis* spores and the efficacy of the spore treating scheme require further research.

In conclusion, our research has developed a new type of *B. subtilis* that expresses mSEB on the surface of its spores. Our results demonstrated that mSEB spores can be safely applied and that they trigger an immune response in the investigated mice. Oral administration of mSEB spores was shown produced partial protective effect though SEB lethal infection.

## Data Availability

Not applicable.
